# Sunlight Exposure and Phototherapy: Perspectives for Healthy Aging in an Era of COVID-19

**DOI:** 10.3390/ijerph182010950

**Published:** 2021-10-18

**Authors:** Toshiaki Nakano, Kuei-Chen Chiang, Chien-Chih Chen, Po-Jung Chen, Chia-Yun Lai, Li-Wen Hsu, Naoya Ohmori, Takeshi Goto, Chao-Long Chen, Shigeru Goto

**Affiliations:** 1 Graduate Institute of Clinical Medical Sciences, Chang Gung University College of Medicine, Kaohsiung 833, Taiwan; kueichenchiang@gmail.com (K.-C.C.); killua13469@gmail.com (P.-J.C.); hsuliwen1230@gmail.com (L.-W.H.); 2 Liver Transplantation Center, Kaohsiung Chang Gung Memorial Hospital, Kaohsiung 833, Taiwan; chenfather@adm.cgmh.org.tw (C.-C.C.); may0313@cgmh.org.tw (C.-Y.L.); clchen@cgmh.org.tw (C.-L.C.); 3 Department of Psychiatry, Kaohsiung Chang Gung Memorial Hospital, Kaohsiung 833, Taiwan; 4 Faculty of Nursing, Josai International University, Togane 283-8555, Japan; nohmori@jiu.ac.jp (N.O.); tgoto@jiu.ac.jp (T.G.); 5 Kazusa Institute for Drug Discovery, Josai International University, Togane 283-8555, Japan; 6 Nobeoka Medical Check Center, Fukuoka Institution of Occupational Health, Nobeoka 882-0872, Japan

**Keywords:** sunlight, phototherapy, vitamin D_3_, alarmins, microbiota, COVID-19

## Abstract

Most humans depend on sunlight exposure to satisfy their requirements for vitamin D_3_. However, the destruction of the ozone layer in the past few decades has increased the risk of skin aging and wrinkling caused by excessive exposure to ultraviolet (UV) radiation, which may also promote the risk of skin cancer development. The promotion of public health recommendations to avoid sunlight exposure would reduce the risk of skin cancer, but it would also enhance the risk of vitamin D_3_ insufficiency/deficiency, which may cause disease development and progression. In addition, the ongoing global COVID-19 pandemic may further reduce sunlight exposure due to stay-at-home policies, resulting in difficulty in active and healthy aging. In this review article, we performed a literature search in PubMed and provided an overview of basic and clinical data regarding the impact of sunlight exposure and vitamin D_3_ on public health. We also discuss the potential mechanisms and clinical value of phototherapy with a full-spectrum light (notably blue, red, and near-infrared light) as an alternative to sunlight exposure, which may contribute to combating COVID-19 and promoting active and healthy aging in current aged/superaged societies.

## 1. Introduction: Impact of Sunlight on Active and Healthy Aging

Sunlight exposure, water, and carbon dioxide are essential for the release of oxygen into the atmosphere and the growth of plants, trees, and their organic products by photosynthesis [1]. Animals can survive by breathing fresh air and eating plant/animal-based foods. In other words, photosynthesis is essential to all life on Earth, including humans. In addition to the role of photosynthesis in the supply of fresh air and food products, there are many benefits of sunlight exposure in our biological activities for active and healthy aging. The first description of the benefits of sunlight exposure was found in a book written by Hippocrates of Kos (460–377 BCE), the father of modern medicine [2]. He described the impact of sunlight exposure on wounds, tetanus, bone fracture, obesity, and mood disorders [3]. In her book *Notes of Nursing: What it is and What it is Not*, Florence Nightingale (1820–1910), the founder of modern nursing, also mentioned that light is one of five essential points for the health of houses, in addition to pure air, pure water, drainage and cleanliness [4].

One of the potential mechanisms underlying the association between sunlight exposure and public health is the biosynthesis of vitamin D_3_, which is known as the “sunshine vitamin.” Most humans depend on sunlight exposure to satisfy their requirements for vitamin D_3_; otherwise, it can be obtained from vitamin D-rich diets (e.g., oily fish, red meat, liver, egg yolks, mushroom) or supplements (e.g., cod liver oil) [5]. Briefly, solar ultraviolet B (UV-B; 280–315 nm) photons are absorbed by 7-dehydrocholesterol (7-DHC) in the skin, leading to its transformation to previtamin D_3_, which is rapidly converted to vitamin D_3_. Once formed, vitamin D_3_ is metabolized in the liver to 25-hydroxyvitamin D_3_ (25(OH)D_3_; calcidiol) followed by conversion into its biologically active form, 1α, 25-dihydroxyvitamin D_3_ (1,25(OH)_2_D_3_; calcitriol), in the kidney [6]. However, too much UV radiation (UV-A; 315–400 nm and UV-B) has reached Earth. UV-A and UV-B contribute to skin aging and wrinkling and promote the development of skin cancer [7], leading to the promotion of many public health recommendations to avoid excessive sunlight exposure. The avoidance of excessive sunlight exposure may reduce the risk of skin cancer, but insufficient sunlight exposure can cause vitamin D_3_ insufficiency, which is associated with many diseases, such as osteoporosis, rickets, psychiatric disorders, infections, allergies, autoimmune diseases, cardiovascular diseases, metabolic syndrome and cancers [8,9,10]. In addition, aging may also affect the formation of 1,25(OH)_2_D_3_ due to age-related reductions of renal function [11]. How to solve this dilemma (benefit vs. disadvantage of sunlight exposure) is an important issue for achieving active and healthy aging in current aged and superaged societies [12,13,14,15]. Furthermore, the current outbreak of COVID-19 has caused worldwide health and economic burdens. Many studies have discussed the association between sunlight exposure and the global COVID-19 pandemic caused by severe acute respiratory syndrome coronavirus 2 (SARS-CoV-2), and vitamin D_3_ has been considered one of the contributing factors for the prevention of COVID-19 [16,17,18,19]. In addition, the direct impact of solar UV-A/-B or artificial UV-C (100–280 nm) radiation on the inactivation of SARS-CoV-2 has been reported [20,21]. UV-C radiation may induce viral genome damage without apparent changes in viral morphology, resulting in the inactivation of SARS-CoV-2 [21].

In this review article, we summarize the impact of sunlight exposure and vitamin D_3_ on public health and identify the risk factors and potential mechanisms of COVID-19 and severe illness. This work is based on a literature search in PubMed until 27 September 2021 using the search terms “sunlight and vitamin D and health [Filter applied: Review] (*n* = 518)”, “sun exposure and public health [found by citation matching] (*n* = 8)”, “vitamin D biosynthesis and aging (ageing) [Filter applied: Review] (*n* = 551)”, “sunlight and COVID-19 (*n* = 301)”, “COVID-19 and UV and latitude (*n* = 6)”, “UV radiation and COVID-19 (*n* = 221)”, “phototherapy and COVID-19 (*n* = 111)”, “founder of phototherapy and dermatology (*n* = 9)”, “phototherapy and psychiatry (Filter applied: Review) (*n* = 162)”, “phototherapy and sleep disorder (*n* = 677)”, “phototherapy and Alzheimer’s disease and dementia (Filter applied: Review) (*n* = 38)”, “ COVID-19 and risk factors and obesity and diabetes (*n* = 645)”, “COVID-19 and vitamin D deficiency (*n* = 369)”, “COVID-19 and angiotensin and ACE2 (*n* = 4708)”, “COVID-19 and angiotensin and ACE2 and vitamin D (*n* = 51)”, “COVID-19 and adipokines (*n* = 38)”, “COVID-19 and alarmins (*n* = 36)”, “angiotensin and alarmins (*n* = 14)”, “COVID-19 and oxidative stress and antioxidants (*n* = 221)”, “COVID-19 and gut dysbiosis (*n* = 124)”, “COVID-19 and fecal (fecal) microbiota transplantation (*n* = 37)”, “sunlight and gut dysbiosis (*n* = 4)”, “visible light and opsins (Filter applied: Review) (*n* = 556)”, “visible light and opsins and lung (*n* = 14)”, “visible light and opsins and adipose tissues (*n* = 6)”, or “opsins and cancer (*n* = 176)”. We then integrate our previous and present data and discuss the potential mechanisms and clinical value of phototherapy with full-spectrum light (notably blue, red, and near-infrared light) as an alternative to sunlight exposure for contributing to active and healthy aging, notably in the era of COVID-19.

## 2. Phototherapy for Active and Healthy Aging: History and Current Applications

The first published application of phototherapy was conducted by Niels Ryberg Finsen (1860–1904), who developed a carbon arc lamp for the treatment of skin tuberculosis (lupus vulgaris), and his clinical contribution in dermatology was awarded the Nobel Prize in Physiology or Medicine in 1903 [22]. The mechanism of action of Finsen’s carbon arc lamp for the treatment of skin tuberculosis has not been fully elucidated, but evidence suggests the impact of violet/blue (400–470 nm) light on antimicrobial activity [23]. In addition to the application of phototherapy in skin diseases, phototherapy has been widely applied for many diseases, including mental disorders [24,25], sleep disorders [26,27], and neurological disorders [28,29]. In addition, phototherapy is suitable for shift workers, such as rotating night-shift hospital workers who have a higher risk of vitamin D_3_ insufficiency/deficiency [30]. Well-designed studies using phototherapy for shift workers have been conducted at the National Aeronautics and Space Administration (NASA), and treatment subjects have reported better sleep, performance, and physical and mental well-being than control subjects due to the adjustment of circadian rhythms [31,32,33].

**Figure 1:**
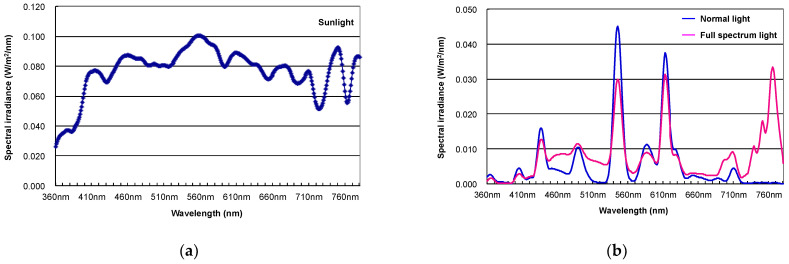
Comparison of the spectrum data in (**a**) sunlight (in September, noon, through the window, exposure value 6500 l×), (**b**) normal light (blue, color rendition index 81, exposure value 780 l×) and (**b**) full-spectrum light (magenta, color rendition index 94, exposure value 750 l×). Spectral irradiance of the light spectrum was measured by an illuminance spectrophotometer (CL-500A, Konica Minolta, Inc., Tokyo, Japan).

**Figure 2:**
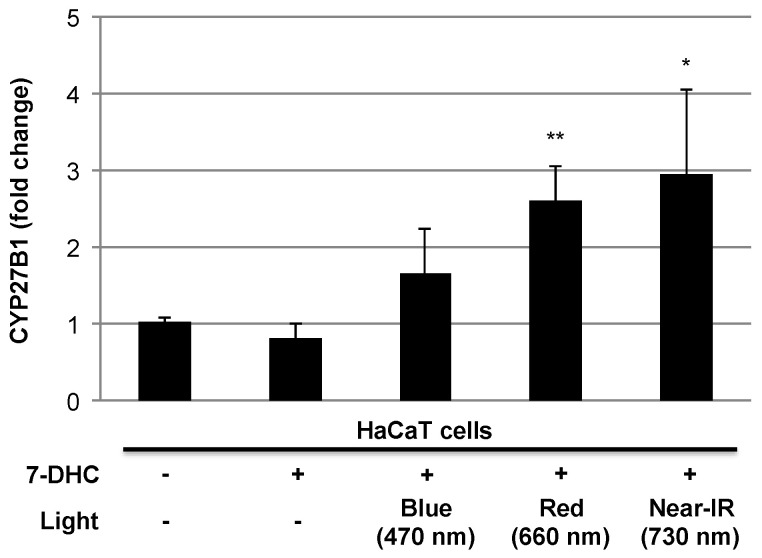
Impact of blue, red, or near-infrared (near-IR) light on the expression of CYP27B1. The human keratinocyte cell line HaCaT was preincubated with 7-dehydrocholesterol (7-DHC, 25 μM) for 24 h, and the cells were irradiated with blue light (470 nm, exposure value 3300 l×), red light (660 nm, exposure value 1600 l×), or near-IR light (730 nm, exposure value 74 l×) for 3 h at 37 °C in 5% CO2/95% air. Twenty-four hours after irradiation, the cells were harvested, and the expression level of CYP27B1 was evaluated by quantitative real-time PCR. Values are presented as the means ± SD of two independent experiments. *, ** *p* < 0.05 and 0.01 vs. control without light irradiation (*n* = 4), respectively (Student’s *t*-test).

In our previous study, we demonstrated lower expression of vitamin D_3_ (calcidiol and calcitriol) in nonalcoholic steatohepatitis (NASH), and phototherapy with full-spectrum light (color temperature 5500 K, color rendition index >90 Ra, distance from animals and light 45 cm, exposure value 600–750 l×, Chang Gung Biotechnology, Taipei, Taiwan) 12 h/day for 6 weeks could elevate vitamin D_3_ levels, resulting in the amelioration of NASH progression in rats [34]. In this study, we demonstrated the altered expression of vitamin D_3_ and lipid transfer/metabolic proteins, such as apolipoprotein E (apoE) and adiponectin, by phototherapy with full-spectrum light [34]. However, the intensities of UV-A (315–400 nm, Figure 1) and UV-B (280–315 nm, [35]) are fairly low and comparable to normal light. UV-B irradiation is indispensable for the photoconversion of 7-DHC to previtamin D_3_ in the skin [36], and a long duration of light exposure (12 h/day) may trigger this reaction both in the control and phototherapy groups. However, the serum levels of calcidiol and calcitriol in the phototherapy group were significantly higher than those in the control group [34]. Interestingly, our preliminary study revealed elevated CYP27B1, which generates active vitamin D_3_ [37] in human keratinocyte HaCaT cells pretreated with 7-DHC (25 μM) for 24 h followed by 3 h irradiation with red (660 nm) or near-infrared light (730 nm) (Figure 2). Therefore, phototherapy with full-spectrum light may play a certain role in vitamin D_3_ metabolism partly through the induction of CYP27B1 for active vitamin D_3_ generation. In support of our observations, a recent review article mentioned the fundamental role of red and near-infrared light in improved health status induced by sunlight exposure [38]. The impact of visible or non-infrared light on vitamin D_3_ biosynthesis should be further explored using full-spectrum light with UV cut-off filters. In addition to the clinical impact of phototherapy in terms of the elevated vitamin D_3_ in NASH patients [35], we and our collaborator have demonstrated the therapeutic potential of phototherapy in experimental animal models of colitis [39] peritonitis [40], and food allergies [41]. Although the mode of action of phototherapy with full-spectrum light has not been fully elucidated, it may regulate proinflammatory cytokine signaling and oxidative stress and maintain optimal levels of vitamin D_3_ and healthy microbiota composition. Interestingly, accumulating evidence suggests the impact of microbiota on circadian rhythms and human health [42]. These evidences suggest the therapeutic potential of phototherapy with full-spectrum light in many diseases associated with vitamin D_3_ insufficiency/deficiency, circadian rhythm disruption and gut dysbiosis.

## 3. Risk Factors and Potential Mechanisms of Severity and Mortality of COVID-19

**Figure 3:**
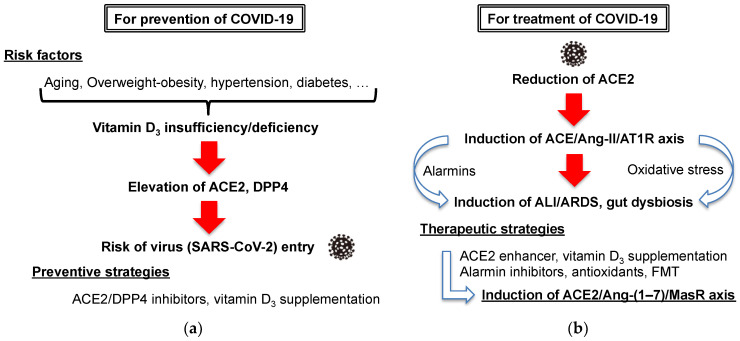
Risk factors and current strategies for the (**a**) prevention and (**b**) treatment of COVID-19 and severe illness. ACE2: angiotensin-converting enzyme 2, ALI: acute lung injury, Ang-(1–7): angiotensin 1–7, Ang-II: angiotensin II, AT1R: angiotensin type I receptor, ARDS: acute respiratory distress syndrome, DPP4: dipeptidyl peptidase 4, FMT: fecal microbiota transplantation, MasR: Mas receptor.

There are many risk factors associated with the severity and mortality of COVID-19, including aging, overweight-obesity, hypertension, diabetes, and lung, cardiovascular, and kidney diseases [43]. In addition, many studies have observed an association between vitamin D_3_ insufficiency/deficiency and COVID-19, suggesting the therapeutic potential of vitamin D_3_ supplementation for the prevention and treatment of SARS-CoV-2 infection [44,45,46,47,48]. One of the potential mechanisms behind SARS-CoV-2 infection is the altered expression of receptors for virus entry, such as angiotensin-converting enzyme 2 (ACE2) and dipeptidyl peptidase 4 (DPP4, also known as CD26), in patients with the mentioned risk factors [49,50,51,52]. Although a recent molecular docking study did not support the effective interaction between DPP4 and SARS-CoV-2 spike protein for virus entry [53], blockade of ACE2 and DPP4 has been proposed as a preventive strategy for COVID-19 [54,55] (Figure 3a). Another possibility is that SARS-CoV-2 infection could reduce ACE2 expression due to attachment of the SARS-CoV-2 spike protein, resulting in induction of the ACE/angiotensin II (Ang-II)/angiotensin type I receptor (AT1R) axis, which is associated with acute lung injury (ALI)/acute respiratory distress syndrome (ARDS). Therefore, the development of drugs that enhance ACE2 activity may be a promising approach for the treatment of COVID-19 and severe illness [56]. In terms of the above renin-angiotensin system (RAS), vitamin D_3_ supplementation could modulate unbalanced RAS and ACE2 downregulation, resulting in induction of the ACE2/Ang-(1–7)/Mas receptor (MasR) axis for protection against ALI/ARDS [57] (Figure 3b).

In addition, adipose tissues are one of the largest endocrine organs and a source of proinflammatory mediators and adipokines, which may create chronic low-grade inflammatory preconditioning [58,59]. Therefore, preexisting chronic inflammation and further inflammatory responses against virus infection lead to extreme systemic inflammation known as a cytokine storm, resulting in the increased severity and mortality of SARS-CoV-2 infection [60]. These COVID-19 patients with poor outcomes have been associated with gut dysbiosis [61]. Interestingly, a recent study pointed to the therapeutic potential of oral Ang-(1–7) peptide in obese mice by modulating the intestinal microbiota (reduction in *Firmicutes*/*Bacteroidetes* ratio), suggesting the involvement of RAS in obesity, gut dysbiosis, COVID-19, and ALI/ARDS [62].

Recent studies have pointed to the significance of early detection of danger signals for the classification of COVID-19 patients as being at high risk of mortality. Alarmins are possible danger signals associated with COVID-19 and comorbidities [63], and S100A8/A9, high mobility group box 1 (HMGB1), and histones are considered potential therapeutic targets [64,65,66]. The elevation of S100A8/A9 and HMGB1 by Ang-II suggests the involvement of alarmins in unbalanced RAS [67,68], and the ACE2/Ang-(1–7)/MasR axis could suppress HMGB1 signaling [69]. Although there is no application of alarmin blockade in COVID-19 and comorbidities, previous studies have suggested the therapeutic potential of neutralizing antibodies against S100A8/A9, HMGB1, or histones for the inhibition of pulmonary fibrosis and sepsis-associated ALI/ARDS [70,71,72,73]. The suppression of oxidative stress is also a potential strategy for the treatment of COVID-19, and our previous study demonstrated the induction of nuclear factor-erythroid 2-related factor 2 (Nrf2), a master regulator of antioxidant responses, such as heme oxygenase-1 (HO-1), superoxide dismutase 1 (SOD1) and SOD2, by phototherapy with full-spectrum light [41]. Some potential antioxidants, such as vitamin C, glutathione, melatonin, and α-lipoic acid, have also been proposed for clinical applications in COVID-19 [74,75,76,77].

## 4. Hypothesis: Potential Impact of Phototherapy with Full-Spectrum Light on the COVID-19 Pandemic

Based on the current understanding of risk factors, prognostic factors, and mechanisms of action of SARS-CoV-2, one of the promising strategies for the prevention of infection and recovery from severe illness may be the maintenance of optimal levels of vitamin D_3_ and the reduction in risk factors. However, a recent randomized clinical trial with a single oral dose of vitamin D_3_ (200,000 IU) did not reduce the hospital length of stay in patients with moderate to severe COVID-19 [78]. Furthermore, a Mendelian randomization study did not reveal evidence to support an association between calcidiol levels and COVID-19 susceptibility, severity, or hospitalization [79]. On the other hand, another randomized clinical trial with daily oral vitamin D_3_ (5000 IU) for two weeks reduced the time to recovery for symptoms such as cough and gustatory sensory loss among mild to moderate COVID-19 patients with suboptimal vitamin D_3_ status [80]. Another large-scale population-based cohort study observed that patients on vitamin D_3_ supplementation who achieved serum 25(OH)D_3_ levels ≥30 ng/mL had a lower risk of SARS-CoV-2 infection, severity, and mortality than unsupplemented controls [81]. These clinical trials suggest that vitamin D_3_ supplementation may be effective for the prevention of SARS-CoV-2 infection and the treatment of symptoms, but deciding the dose and duration of vitamin D_3_ supplementation must be an important point for achieving better COVID-19 outcomes. Most importantly, misuse of vitamin D_3_ supplementation may rarely cause vitamin D intoxication, leading to hypercalcemia and serious kidney, heart, and neurological problems [82,83]. On the other hand, there is no risk of vitamin D intoxication even through excessive exposure to sunlight [84].

**Figure 4:**
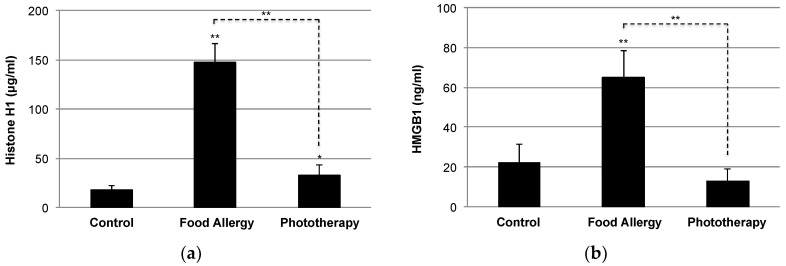
Elevation of circulating alarmins during inflammatory responses. BALB/c mice (*n* = 12) were sensitized with ovalbumin (OVA)/alum followed by intragastric ingestion of OVA for the development of a mouse model of food allergy (FA) [35]. Circulating levels of (**a**) histone H1 and (**b**) high mobility group box 1 (HMGB1) were quantified using an enzyme-linked immunosorbent assay (ELISA) as previously described [87,88]. Values are presented as the means ± SD of four to six individuals in each group. Phototherapy with full-spectrum light (*n* = 6) ameliorated FA-like allergic diarrhea in FA mice (*n* = 6), with significant suppression of circulating histone H1 and HMGB1 released from damaged cells or actively secreted from immune cells (e.g., macrophages, dendritic cells, mast cells). *, ** *p* < 0.05 and 0.01 vs. control (*n* = 4) or phototherapy group, respectively (Student’s *t*-test).

**Figure 5:**
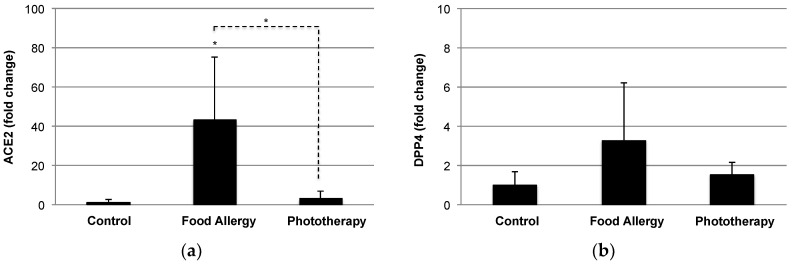
Elevation of (**a**) angiotensin-converting enzyme 2 (ACE2) and (**b**) dipeptidyl peptidase 4 (DPP4) in inflamed colon tissues. Colon tissues were obtained from naïve mice (*n* = 4), food allergy (FA) mice (*n* = 4), and FA mice with phototherapy (*n* = 6) [35], and colonic levels of ACE2 and DPP4 were evaluated by quantitative real-time PCR. Values are presented as the means ± SD of four individuals in each group. Although there was a large variation in expression profiles due to the different intensities of inflammation in each FA mouse, we confirmed the tendency to increase colonic levels of ACE2 and DPP4, key receptors for SARS-CoV-2 entry, in FA. On the other hand, phototherapy with full-spectrum light suppressed these expression levels, suggesting the preventive potential of phototherapy in COVID-19. * *p* < 0.05 vs. control or phototherapy group (Student’s *t*-test).

Although there is no direct evidence that phototherapy could prevent or ameliorate SARS-CoV-2 infection or COVID-19 comorbidities, previous and present observations have suggested the potential of phototherapy with full-spectrum light in COVID-19. Furthermore, there are several advantages of phototherapy with full-spectrum light for the prevention and treatment of COVID-19. First, phototherapy with full-spectrum light would be a safe strategy to satisfy vitamin D_3_ without the risk of vitamin D intoxication because it would be expected to generate sufficient vitamin D_3_ under the appropriate exposure regimen (12 h/day for 6–9 weeks) [34,41]; i.e., a single exposure would not produce sufficient vitamin D_3_, and multiple standard-dose exposures (exposure value 600–750 l×) over a period of time would be required. Second, phototherapy with full-spectrum light would ameliorate the adipose tissue dysfunction, which causes insulin resistance, proinflammatory cytokine release, and altered adipokine production [34]. Reduced expression of adiponectin is a risk factor of metabolic syndrome, and a recent case-control study pointed to the link between obesity and COVID-19 respiratory failure in terms of adiponectin levels [85]. Our previous study demonstrated the elevation of adiponectin by phototherapy with full-spectrum light [34]. A recent study also pointed to the involvement of apoE in virus (SARS-CoV-2) entry by hijacking the metabolic pathway of apoE [86]. Third, phototherapy with full-spectrum light reduces circulating levels of alarmins, such as histone H1 and HMGB1 (Figure 4), which reflect the severity of inflammatory responses [35]. A similar elevation of alarmins was confirmed in septic mice [71,72] and in rats undergoing rejection [87,88], resulting in local and systemic inflammation. The suppression of oxidative stress by the Nrf2-mediated antioxidant response may be a potential mechanism of phototherapy with full-spectrum light. Fourth, phototherapy with full-spectrum light can improve gut dysbiosis by modulating the *Firmicutes*/*Bacteroidetes* ratio [41]. Elevation of the *Firmicutes*/*Bacteroidetes* ratio was reported in COVID-19 patients and was reduced in the recovery state [89]. In our recent study, we identified the genus *Lachnospiraceae_NK4A136_group* (phylum *Firmicutes*) as a food allergy-associated bacteria [41], and a recent study pointed to the existence of gut-associated bacteria, such as the family *Lachnospiraceae* in the lung microbiota of patients with ARDS [90]. Interestingly, recent studies introduced the therapeutic potential of fecal microbiota transplantation (FMT) for recurrent *Clostridium difficile* infection patients with COVID-19 [91,92]. In addition, a clinical trial (NCT04824222) to assess the impact of FMT on reducing the risk of disease progression as a supplement to standard COVID-19 treatment is ongoing [92]. On the other hand, *Parabacteroides goldsteinii* (phylum *Bacteroidetes*) was identified as a beneficial bacterial species enriched by phototherapy with full-spectrum light [41]. Gut commensal *Parabacteroides goldsteinii* plays an important role in the anti-obesity effect of polysaccharides isolated from *Hirsutella sinensis* [93], a traditional Chinese medicine known to possess various pharmacological properties, including the attenuation of pulmonary inflammation and fibrosis [94]. Recently, the same group demonstrated the prevention of chronic obstructive pulmonary syndrome (COPD) by lipopolysaccharide derived from *Parabacteroides goldsteinii* [95], suggesting the therapeutic potential of phototherapy with full-spectrum light in COVID-19 in part through the induction of beneficial bacteria, such as *Parabacteroides goldsteinii*. Finally, our preliminary data suggest the impact of phototherapy with full-spectrum light on altered expression of ACE2 and DPP4 (CD26), receptors for SARS-CoV-2 entry in inflamed colon tissues (Figure 5). Due to the large distribution of ACE2 and DPP4 (CD26) in the human body, SARS-CoV-2 may infect other tissues aside from the lungs [50,96], and diarrhea is a common presenting symptom in COVID-19 patients [97].

**Figure 6:**
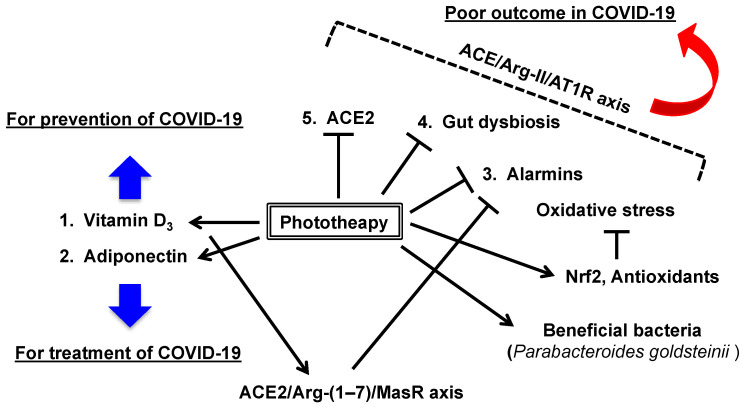
Proposed mechanisms of phototherapy with full-spectrum light for the prevention and treatment of COVID-19. Phototherapy may induce (1) vitamin D_3_ biogenesis and (2) adiponectin, which are associated with both prevention and treatment of COVID-19 in terms of the reduction in COVID-19 risk factors (Figure 1). (3) The elevation of circulating alarmins and oxidative stress markers during inflammatory responses may be inhibited by phototherapy, partly through the induction of Nrf2-mediated antioxidant responses. (4) Gut dysbiosis (an elevated *Firmicutes*/*Bacteroidetes* ratio) may be modulated by phototherapy and may induce the beneficial bacteria *Parabacteroides goldsteinii*. (5) Phototherapy may reduce ACE2 expression, resulting in a reduced risk of virus (SARS-CoV-2) infection. For treatment, vitamin D_3_ may induce the ACE2/Ang-(1–7)/MasR axis and inhibit Ang-II-induced alarmin elevation, oxidative stress responses, and gut dysbiosis, which are associated with poor outcomes in COVID-19. ACE2: angiotensin-converting enzyme 2, Ang-(1–7): angiotensin 1–7, Ang-II: angiotensin II, AT1R: angiotensin type I receptor, MasR: Mas receptor, Nrf2: nuclear factor-erythroid 2-related factor 2.

Taken together, phototherapy with full-spectrum light induces vitamin D_3_ biosynthesis, alters adipokine production (elevated adiponectin), modulates microbiota composition (reduction in the *Firmicutes*/*Bacteroidetes* ratio and induction of beneficial bacteria, i.e., *Parabacteroides goldsteinii*), and reduces many risk factors (e.g., alarmins, proinflammatory cytokines, and oxidative stress markers) associated with COVID-19 and severe illness (Figure 6).

## 5. Summary and Future Directions for Active and Healthy Aging

**Figure 7:**
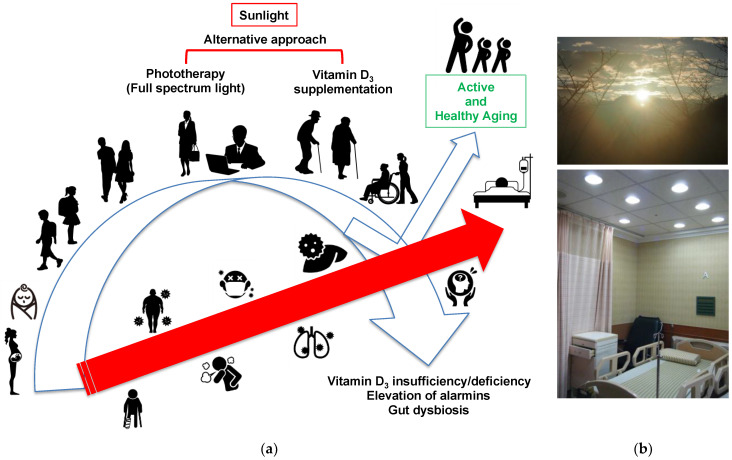
Roadmap for active and healthy aging. (**a**) Sunlight exposure is fundamental for our health, including infants to elderly individuals. Vitamin D_3_ levels decline in the aging process as well as due to insufficient sunlight exposure and stay-at-home policies in the era of COVID-19, resulting in an increased risk of various diseases (red arrow) associated with vitamin D_3_ insufficiency/deficiency, elevation of circulating alarmins, and gut dysbiosis. In addition to sunlight exposure and vitamin D_3_ supplementation, phototherapy may maintain the vitamin D_3_ concentration required for biological, physiological, and immunological activities, resulting in the achievement of active and healthy aging. (**b**) Sunlight and phototherapy as an alternative to sunlight exposure. A ward equipped with full-spectrum light (color temperature 5500 K, color rendition index >90 Ra, Chang Gung Biotechnology, Taipei, Taiwan) was used for the clinical application of phototherapy.

Sunlight exposure and appropriate exercise may be the best ways to maintain healthy conditions. However, vitamin D_3_ biosynthesis depends on the strength of UV radiation, its exposure time as well as skin color (the type of melanin) [98,99]. Therefore, optimal conditions for vitamin D_3_ biosynthesis may be different in each individual, and seasonal changes in sunlight exposure may also affect it. We also need to consider the adverse effects (skin aging, wrinkling, and skin cancer development) caused by excessive UV exposure. In addition, many people, such as night-shift workers, elderly people with difficulty walking, and people who are hospitalized, may be limited to receiving enough benefits from sunlight. In current aged/superaged societies and an ongoing global pandemic of COVID-19, we need to consider ways to enhance our immunity to maintain suitable health. Notably, the stay-at-home policy may reduce the time for outdoor activities, resulting in vitamin D_3_ insufficiency/deficiency [100] and an increased incidence of bone fractures in the elderly [101]. In addition to sunlight exposure and vitamin D_3_ supplementation, phototherapy with full-spectrum light is an alternative approach to stimulate vitamin D_3_ biosynthesis with minimal risk of skin damage and adjust circadian rhythms, which are quite important for biological, physiological, and immunological activities in all living organisms. To achieve healthy aging, the age-dependent decline in vitamin D_3_ biosynthesis should be actively adjusted by vitamin D_3_ supplementation and phototherapy with full-spectrum light, which may reduce the risk of various diseases, such as osteoporosis, metabolic syndromes, allergies, infectious disorders, mental/neurological disorders and cancers (Figure 7a). In the case of vitamin D_3_ supplementation, we may need to carefully evaluate the optimal dose of vitamin D_3_ to reduce the risk of vitamin D intoxication and relative complications. To further explore the clinical impact of phototherapy with full-spectrum light, we set up a phototherapy room for patients (Figure 7b). Although we have no data to demonstrate the involvement of vitamin D_3_, we have confirmed some beneficial effects of phototherapy, such as the reduction in total bilirubin in a patient with severe jaundice after liver transplantation (data not shown). Although further small and large cohort studies are necessary, phototherapy with full-spectrum light could be a reasonable approach with a low potential risk of adverse events, and we highly recommend changing the indoor light environment to full-spectrum light at homes and public spaces, such as schools, working places, clinics, and hospitals as well as nursing homes. The cost of full-spectrum light (roughly three times higher than normal light) may be a critical issue, but there are many beneficial impacts of phototherapy with full-spectrum light, including the generation of sufficient vitamin D_3_ and the maintenance of healthy gut microbiota composition as well as suitable circadian rhythm. All of these are indispensable for achieving active and healthy aging in current aged/superaged societies.

## 6. Conclusions

In conclusion, we propose phototherapy with full-spectrum light as one of the potential strategies to prevent disease progression associated with vitamin D_3_ insufficiency/deficiency, and it may lead us to achieve active and healthy aging in the era of COVID-19.

Recently, the impact of blue light (450, 454, and 470 nm) on SARS-CoV-2 inactivation was reported [102]. Furthermore, a recent case report suggested the therapeutic potential of phototherapy with a red light (630 + 660 nm) for the alleviation of respiratory symptoms, pulmonary inflammation, and hypoxia [103]. The beneficial effect of red light (660 nm) was also confirmed in an experimental model of sepsis-associated ALI [104]. Although most peripheral tissues, except the eye and skin, are not normally reached by light, accumulating evidence suggests the direct and indirect impacts of visible light on cell behavior and biological activities through opsin (OPN) receptors [105]. For example, OPN3 and OPN4 are expressed in the aorta and pulmonary arteries, and blue light exposure induces vasorelaxation [106,107]. The expression of OPN3 and OPN4 in airway smooth muscle [108] also suggested the impact of blue light exposure on vasorelaxation for the treatment of pulmonary disorders caused by COVID-19. On the other hand, blue light exposure has been shown to suppress melatonin, resulting in a negative impact on sleep quality [109]. In addition, the impact of light on adipose tissues and lipid homeostasis has been reported. Briefly, specific wavelength, especially green light (505 nm), enhanced OPN2 expression in mature 3T3-L1 adipocytes and decreased lipid droplets [110]. Ondrusova K et al. demonstrated the expression of OPN3 in subcutaneous white adipose tissues, and daily exposure of differentiated 3T3-L1 adipocytes to blue light resulted in decreased lipid droplet size [111]. A recent study demonstrated the expression of OPN3 in brown adipose tissues and its impact on the regulation of glucose metabolism and mitochondrial respiration in brown adipocytes [112]. The direct exposure of brown adipose tissues to white light (465 + 565 nm) increased thermogenic capacity in an OPN3-dependent manner, suggesting the potential of phototherapy for obesity and obesity-associated metabolic disorders [112]. Furthermore, the *OPN1SW* (opsin 1, shortwave sensitive), *OPN2*, *OPN3*, and *OPN4* genes are widely found and differentially expressed in human brain areas and potentially regulate the circadian photoentrainment of the central biological clock [105]. The impact of OPN3 or OPN4 on tumor cell activities, such as drug sensitivity, growth, and metastasis, has been reported in hepatocellular carcinoma, colon cancer, and lung adenocarcinoma [113,114,115,116], and blue light (465 nm) exposure suppresses tumor growth by inducing autophagy [114]. Taken together, these observations suggest the possible broad impacts of phototherapy with full-spectrum light on biological, physiological, and immunological activities in multiple organs, tissues, and cells.

Further investigations, including the screening of optimal conditions of phototherapy, such as light strength, effective wavelength, and the duration of exposure, should be considered in future preclinical and clinical trials.

## Data Availability

No datasets were generated or analyzed during the current study.
